# Sustainability considerations in remediation, retrofit, and seismic upgrading of historic masonry structures

**DOI:** 10.1007/s11356-021-17490-7

**Published:** 2021-11-19

**Authors:** Marco Corradi, Enea Mustafaraj, Emanuela Speranzini

**Affiliations:** 1 Department of Mechanical and Construction Engineering, Northumbria University, Newcastle upon Tyne, NE1 8ST UK; 2 Department of Engineering, University of Perugia, 06125 Perugia, Italy; 3 Department of Civil Engineering, EPOKA University, 1032 Tirana, Albania

**Keywords:** Masonry, Timber, Retrofit, Seismic upgrade, Sustainable solutions

## Abstract

This paper addresses the problem of sustainability in remediation, retrofit, and seismic upgrading of historic masonry structures. Different rehabilitation techniques and some successful applications throughout the Balkans and Italy are described, with particular emphasis to the shear reinforcement of wall panels. The selected techniques aim at improving the seismic performance, preserving the structures for future generations, having the least impact in altering the architectural and heritage values, as well as being sustainable, in terms of reduced carbon dioxide emissions, reversibility, and low energy consumption. The use of cross-laminated timber (CLT), natural fibers, and fiber-reinforced Polymers (FRP) jacketing with natural lime coatings are discussed. The paper concludes by summarizing key successes of the proposed rehabilitation solutions in conservation engineering and suggests areas in which these could be used with great advantage.

## Introduction

The largest part of existing building stock in the world is made of stone and brickwork masonry. These structures have often been constructed many years ago using traditional technologies, mostly realized by the rules of common practice relying solely on masters’ knowledge, and often do not comply with the modern design standards (Faella et al. 2010). Masonry buildings have good resistance against gravitational loads but have demonstrated poor performance when subjected to lateral loads induced by the earthquakes.

During earthquakes, depending upon the direction of the seismic wave, the load-bearing walls of a building are subjected to in-plane shear forces and/or out-of-plane bending. The out-of-plane capacity can be increased by improving the wall-to-wall and wall-to-floor connections. By doing this, it is possible to “transfer” the seismic forces from face-loaded walls to the return walls. The in-plane failure in the form of shear failure, sliding failure, rocking failure, or toe crushing failure is considered to be more dangerous and needs to be addressed properly (Magenes and Calvi 1997; Wood 1998; Kalali and Kabir 2012; Gattesco and Boem 2015).

However, increasing the structural safety of old masonry buildings is a challenging task: for many years, the solution adopted by governments and international bodies was to demolish these old masonry structures, promoting the construction of new buildings; highly increasing water and energy consumption, waste, and land take from agriculture; and putting at risk the natural environment and the fauna. Apart from being a non-sustainable solution, increasing dioxide emissions and energy consumption, demolition and new construction also cause an irreparable loss of cultural heritage. In the last decade, the increased awareness of the importance of masonry preservation due to the values old buildings possess has turned to development of sustainable strategies for retrofit and seismic upgrade of these masonry structures.

Traditional retrofitting techniques such as (i) filling cracks by grouting; (ii) stitching of large cracks with metallic or brick elements; (iii) external or internal post-tensioning with steel ties; (iv) shotcrete jacketing; and (v) ferrocement (Calderini et al. 2010; Padalu et al. 2020; Ma et al. 2021; Dauda et al. 2021) are deemed to be particularly invasive and are not recommended for historic structures. The conservation bodies request structural efficient methods that are less intrusive.

In the recent years, innovative techniques consisting of using fiber-reinforced polymers (FRPs) as well as fabric-reinforced cementitious matrix (FRCM) have been developed. Both methods have been proven to be effective in terms of the increase of capacity and ductility of masonry members without overloading the structure with additional weight (Valluzzi et al. 2002; Gattulli et al. 2014).

Nevertheless, FRP systems, when used with epoxy resins, despite their advantages in the overall structural performance, are considered to be less sustainable due to low fire resistance, high sensitivity to ultraviolet light when exposed to open air, high toxicity, and unsatisfactory long-term behavior (Türk, 2013).

FRCMs on the other hand, combine a fiber-reinforced grid embedded in a high-performance sprayable cementitious matrix and overcome most of the disadvantages coming from the use of the epoxy resins as a binding agent. Apart from these modern materials, the use of organic materials such as natural fibers and timber will be also discussed, with particular emphasis on their “green” properties and suitability characteristics.

## Sustainability in masonry conservation engineering

The principles of sustainability can also be efficiently applied in the conservation and reinforcement of historic masonry buildings (Righetti et al. 2016; Menna et al. 2016; Belliazzi et al. 2018; Misseri et al. 2019). However, several aspects need to be considered, with respect to the materials and methods in which traditional masonry constructions have been built in the past and the hazard to which these buildings are exposed (Bingel and Bown 2009; Sanz et al. 2012; Ferreira et al. 2017).

In addition, an important role needs to be given not only to the climatic conditions of each country where the problem of sustainability of heritage constructions is considered, but also to the attitudes and values of the local national societies, the degree of economic development, and the level of exposure to natural hazards (not only seismic risk, but also the effects of climate change, flooding, and other natural disasters). All these parameters may highly vary across the world (Tomaževič and Lutman 2007; Goodwin et al. 2009; Sassu et al. 2017).

In southern and eastern Europe, where the seismic hazard is significant, a key issue in terms of sustainability is to reduce the emissions of carbon dioxide (CO_2_) and energy use for heating and cooling, while increasing the structural safety of old historic buildings (Straube and Schumacher 2007; De Berardinis et al. 2014; Ruparathna et al. 2016). It is worth citing here the state aid activated in 2021 by the Italian Government for those interventions aimed at reducing both the seismic risk and the energy consumption of existing buildings (“superbonus” program): a tax credit of 110% of all incurred costs is provided to landlords doing such interventions. This law is only one example of a wider agenda aimed at promoting sustainable interventions on the existing building stock, which has major political, economic, and social implications (Italian Act 2020).

For historic buildings, the reduction of carbon dioxide emissions highly depends on the efficiency of buildings. However, there are many more considerations around the sustainable characteristics of masonry conservation: the re-use and conservation of pre-existing masonry structures highly reduce the need for new constructions, and this clearly means a smaller use of material assets for new constructions and reduces transport demand as old historic constructions are typically less dispersed as they are typically more nucleated (in historic centers, medieval villages, and hamlets), while it is well accepted that new constructions, under the typical form of a scattered urban settlement, cause a dramatic increase in transport demand and fossil fuels.

In addition to this, it could also be mentioned that re-use and conservation of historic buildings avoid land take from agriculture for new constructions, reduce the use of construction materials (soils, aggregates, water, etc.), facilitate the preservation of our natural and man-made historic landscapes, and preserve and protect flora and fauna (Wallace et al. 1999; Rodwell 2003; Godwin 2011; Stubbs and Makaš, 2011).

While the advantages of a sustainable conservation of old masonry constructions are evident, how to achieve this is a challenging task. One important consideration, often underestimated in the past, is the need to prevent damaging the heritage and architectural character of the historic buildings where interventions are designed.

The significance of the historic buildings has been often affected by “invasive” retrofit interventions aimed at reducing the seismic risk and/or the carbon dioxide emissions, without considering the heritage values (Borri and Corradi 2019).

**Fig. 1:**
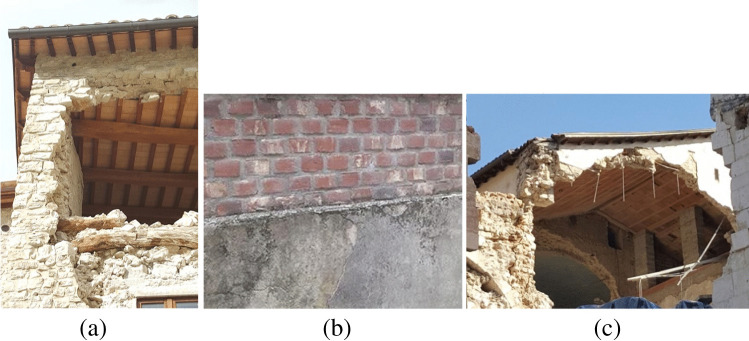
Examples of “invasive” and incorrect seismic interventions: **a** the addition of a reinforced concrete ring beam; **b** the application of a thick, heavy, and stiff coating of reinforced concrete; **c** the replacement of the timber-beam floor with a RC one

Figure 1 shows different incorrect seismic interventions applied in the recent past in many parts of Europe. Figure 1a illustrates the effects of the addition of a reinforced concrete ring beam which was a common type of intervention in the 1980s in Italy, aimed at preventing out-of-plane collapse mechanisms, but recent seismic events have demonstrated that a stiff and heavy reinforced concrete beam may highly facilitate the disaggregation of the underlying masonry during an earthquake (D'Ayala and Speranza 2003; Valluzzi et al. 2004). Figure 1b shows the incorrect use of cement mortars for coating brickwork walls (i.e., difficult to be removed (irreversible) and incompatible with traditional masonry materials), and Fig. 1c shows the effects of the replacement of timber floors with concrete ones, where the increase in mass may produce destructive effects during earthquakes.

**Fig. 2:**
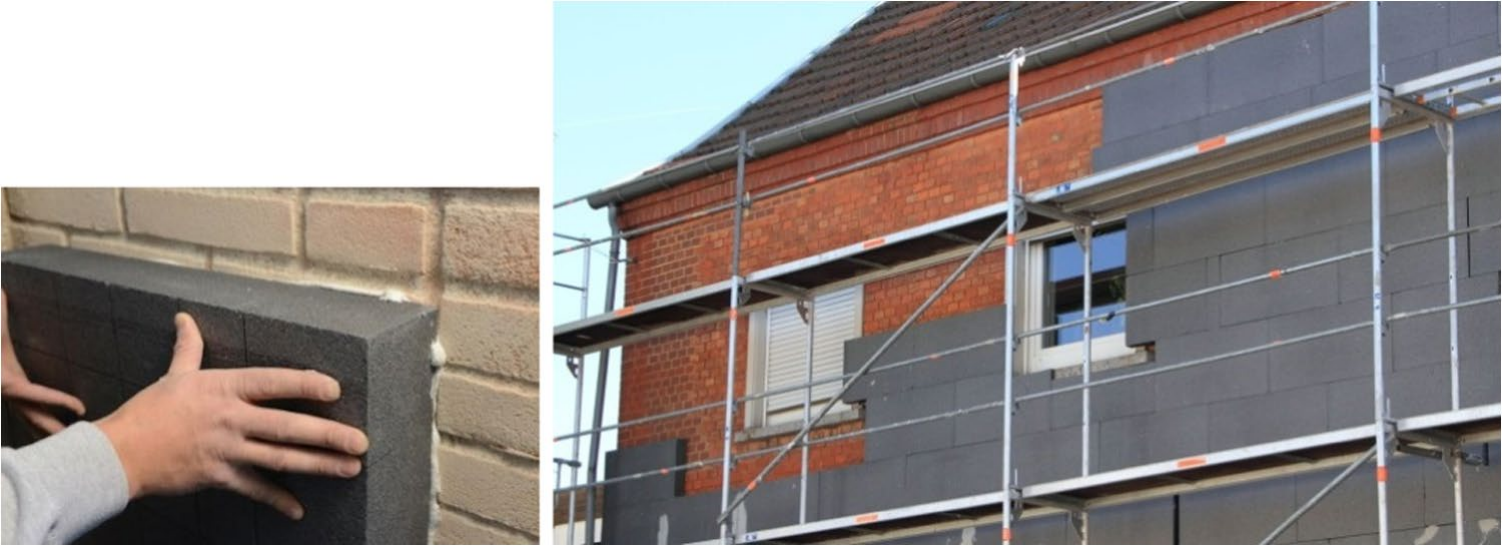
Examples of “invasive” interventions aimed at reducing carbon emissions: the insulation coating covers the historic brickwork masonry and its architectural decorations

The challenge is to improve both the thermal and seismic performances, without affecting the historic significance of the buildings, also using sustainable materials. This is possible through a sensitive adaptation. Figure 2 shows an invasive solution aimed at reducing energy consumption: the insulation coating covers the architectural significance of the historic building. New technical solutions, materials, and methods have recently been proposed, and this paper will summarize these in the following. The aim is preserving local distinctiveness of the historic masonry building stock while achieving the objectives in terms of sustainability and structural safety (Forster 2010; Lagomarsino and Cattari 2015; Anzani et al. 2018).

The process is clearly a multi-disciplinary task, where different competences and skills are involved (structural and materials engineers, architects, drafting specialist, sociologists, conservators, historians, etc.). It can only be achieved by drawing a balance between making alterations to improve thermal and seismic performance, use of sustainable materials (wood, natural fibers, etc.), implementation of cost-effective solutions, and safeguarding the heritage and architectural value of a building.

This is of a paramount importance, especially in seismic prone areas where masonry buildings often present historical and cultural significance. In Italy, an increasing public awareness to these issues has facilitated research in academia and on-site applications. This paper will summarize these solutions, also describing some recent case study sites. It will be demonstrated that, before considering demolition and re-construction of old buildings, it is often possible to achieve satisfactory results in terms of sustainability, structural safety, and preservation of heritage values by adopting solutions able to re-use old buildings.

## Materials and methods

### FRCM

FRCM technique is often considered to be a “green solution” as it incorporates the use of fiber-based meshes embedded in an inorganic (i.e., cementitious or lime) coating, rather than an epoxy resin which is used as a matrix for the FRP systems. FRCM acronym was first used in the American ACI 549.4R-13 (2013) and the Italian Standard CNR-DT 215 (2018), but in literature is known with other names as well, such as textile-reinforced mortar (TRM) (García et al. 2010), fiber-reinforced mortar (FRM) (Cascardi et al. 2017), and mineral-based composites (MBC) (Di Ludovico et al. 2010).

**Fig. 3:**
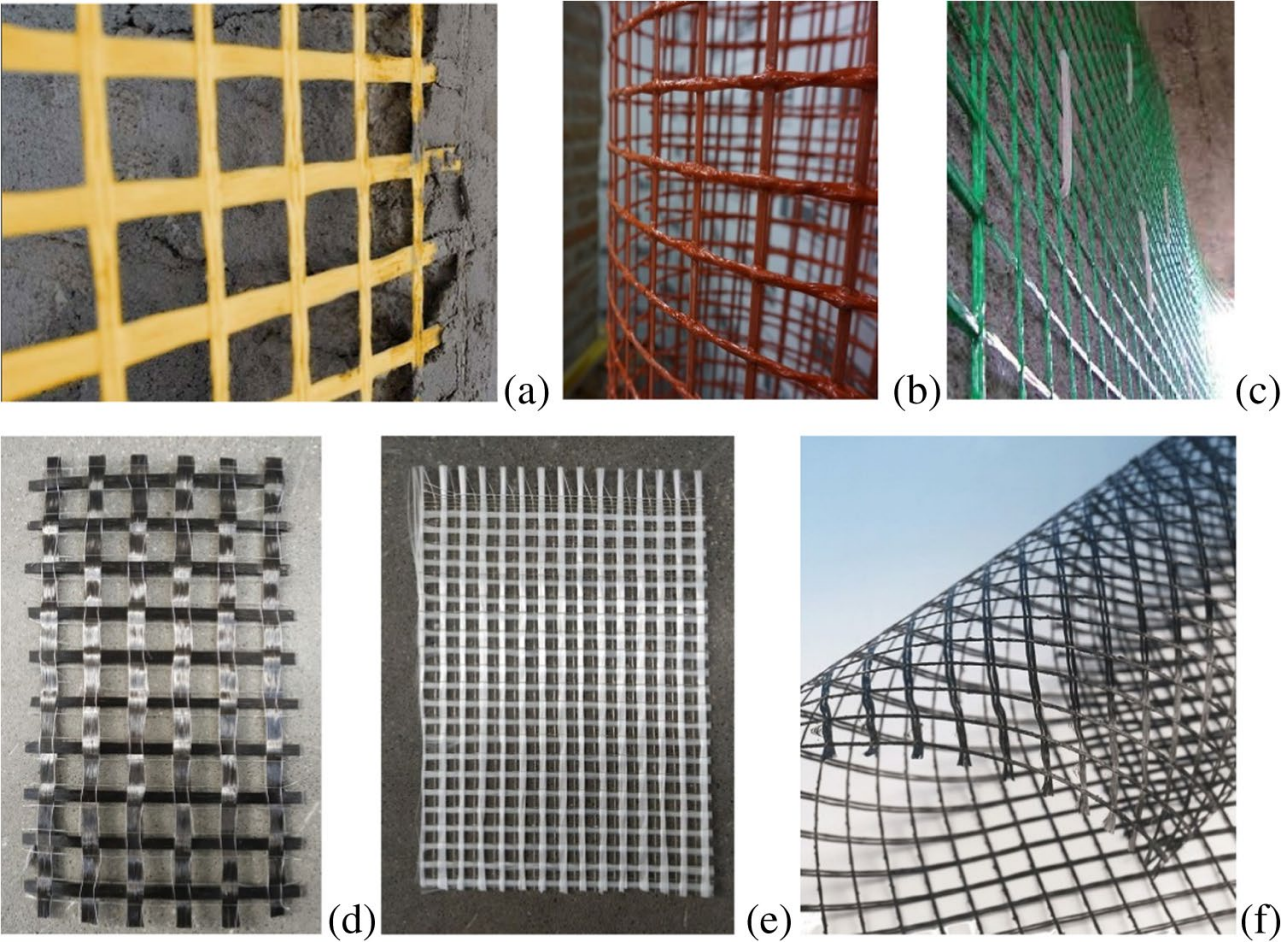
Different types of composite nets and textiles, used in FRCM (**a**, **d**, **e**, **f**) and CRM (**b**, **c**)

**Fig. 4:**
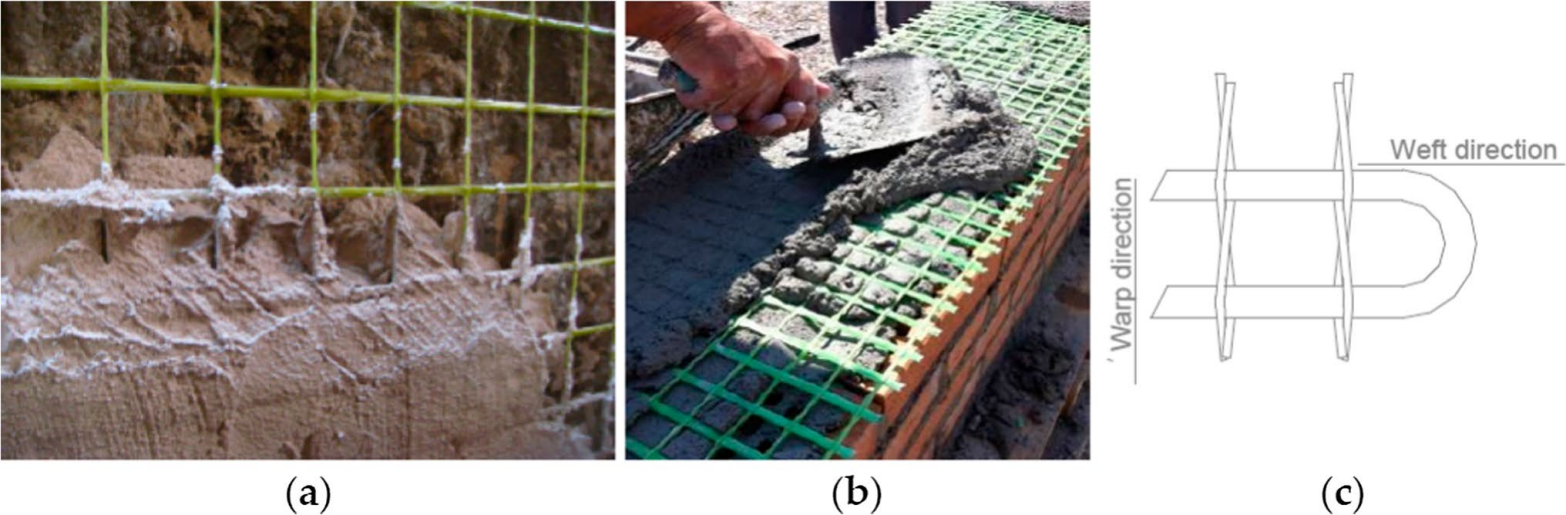
Installation method of FRCM system: **a** and **b** the grid is embedded using a gauging or tiger trowel; **c** the grid is typically made by interlacing two sets of yarns—the weft and the warp

The FRCM system typically consists of a bidirectional textile mesh (mesh size varying between 5 and 50 mm) of basalt (B), carbon (C), glass (G), aramid (A), phenylene-benzo-bisthiazole (PBO), and steel (S) fibers embedded in a cement or lime mortar coating modified with additives (polymers, fly ash, silica fume, or short fibers). The spacing among the mesh facilitates grip of the mortar coating to the fibrous reinforcement and “breathing” of the reinforced walls, i.e., air flowing out through the walls to reduce the concentration of moisture and humidity (Donnini 2019; Napoli and Realfonzo 2020) (Figs. 3 and 4). The denomination of composite-reinforced mortar (CRM) is typically used for rigid meshes embedded in thick (40–60 mm) coating, while FRCM is used for flexible meshes embedded in thin (10–20 mm) coatings (D’Antino et al. 2019, 2020).

The installation process requires a simple preparation of the masonry substrate by removal of previous mortar coating and inconsistent material. Then, a thin layer (5–20 mm) of the mortar coating is applied on the masonry surface of the wall to be strengthened, followed by the installation of the fibrous mesh which is then plastered with a second finishing layer of the mortar (Angiolilli et al. 2020; Mercedes et al. 2020; Mustafaraj and Yardim 2017, 2019). The FRCM system sets and hardens in a few hours and achieves its full strength after 28 days. Table 1 shows a summary of the enhancement of the lateral capacity of shear walls retrofitted with FRCM materials: it can be noted that a significant increment of the structural performance (lateral load capacity, ductility, shear stiffness, deformation capacity) has been demonstrated (Papanicolaou et al. 2011; Corradi et al. 2014; Gattesco and Boem 2015; Borri et al. 2016; D’Antino and Papanicolaou 2017; Younis and Ebead 2018; Monaldo et al. 2019; Longo et al. 2019, 2020). Apart from improving the structural safety under seismic events, FRCMs allow efficient re-use of existing building stock, with evident advantages in terms of sustainability and reduction in use of raw materials. In addition, this retrofitting method is easily removable, if needed, with high degree of reversibility (ICOMOS chapter 2003). The use of an inorganic matrix (lime or cement) has very positive characteristics in terms of “compatibility” between old masonry materials (lime, brick, stone) and the new ones.

**Table 1:** Previous research using FRCM as a strengthening technique for shear walls

Authors	Type of strengthening	Type of masonry	Improvement in lateral load capacity
Angiolilli et al. (2020)	Glass FRCM systems applied as coating to the masonry walls	Stone	420% (620% increase in shear modulus)
Mercedes et al. (2020)	Hemp FRCM	Brickwork	286%
	Cotton FRCM	Brickwork	300%
	Glass FRCM	Brickwork	264%
Mustafaraj and Yardim (2017)	GFRP embedded in cementitious matrix	Brickwork	127% (650% in shear modulus)
Mustafaraj and Yardim (2019)	PP-reinforced mortar coating (repaired after initial failure)	Brickwork	193–255%
Younis and Ebead (2018)	Carbon FRM for strengthening of RC beams	Reinforced concrete beam	200%
	Glass FRM for strengthening of RC beams	Reinforced concrete beam	162%
Corradi et al. (2014)	GFRP grid embedded in lime matrix	Stone Brickwork	400–1060% 330%
Gattesco et al. (201)5	GFRP grid embedded in cementitious matrix		
Papanicolaou et al. (2011)	Carbon FRCM embedded in cementitious matrix Basalt or glass FRCM	Brickwork Stone	> 400% 12–32%
Borri et al. (2016)	GFRP grid embedded in lightweight matrix	Brickwork	7–117%

**Fig. 5:**
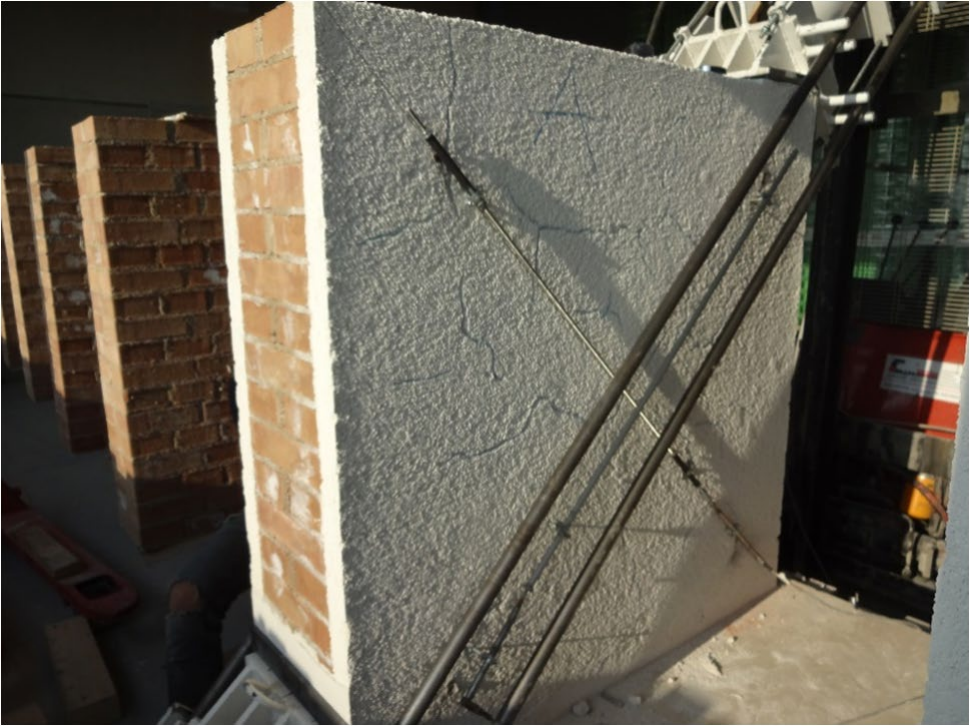
FRCM reinforcement using lightweight mortar mixes: to increase the thermal resistance of the wall, the thickness of the coating needs to be enlarged (typically 100 mm). The weight density of the mortar, used for the coating, can be reduced up to 800 kg/m^3^ without significantly affecting the strengthening effect of the FRCM (Borri et al. 2016)

FRCMs are typically applied on both faces of the walls (Fig. 5). The 2 layers of reinforcement are normally connected using pass-through composite rods: this also produces a positive confinement effect on the reinforced walls able to increase the compressive strength of the masonry.

Another interesting aspect is the energy efficiency of the inorganic matrix in terms of thermal insulation, when this is lightweight or the coating is thick. The plaster layer of a varying thickness of 10–60 mm provides itself an additional layer of insulation, and this can be significant if the depth is sufficiently thick. In order to properly meet the energy requirements of the modern building codes, further analysis is necessary, but first experimental results available in literature confirmed this. Longo et al. (2019) compared the effectiveness of the FRCM technique in terms of mechanical properties as well as energy properties. The FRCM system was designed in such a way that sand used for the mortar coating was replaced with recycled material as waste rubber tires. However, test results were unsatisfactory: it was recorded a fairly small increase in thermal resistance and a significant decrease of the lateral load capacity of the reinforced walls.

This problem could be solved using geo-polymers to form a new FRCM system by combining fly ash binder and expanded glass aggregate (recycled material). The results have a 125% increase in shear strength and a 25% reduction in heat transfer (Longo et al. 2020).

Recently, Borri et al. (2016) investigated the use of thermo-insulating lightweight coatings: while the enhancement in lateral capacity is typically smaller compared to standard cement coating, it was demonstrated that it is possible to find a balance between the need to reinforce and reduce energy consumption by increasing insulation. The increased thickness of the coating (100 mm) and usage of lightweight mortar mixes (weight specific density 800–1000 kg/m^3^) highly improved the thermal resistance of the walls leading towards significant reductions in energy consumption and emissions (Fig. 5).

#### Natural fibers

In more recent years, natural fiber composite materials have sparked the interest of the scientific community and have found application in the reinforcement of old wooden structures as well as masonry. The coupling of masonry with tensile-resistant composite materials is particularly interesting because it gives more structural strength and stiffness, if compared with the performance that masonry can provide alone. Old brick or stone masonry made with lime mortars is particularly weak when loaded in tension and shear, and it is often assumed to have zero tensile strength in design.

**Fig. 6:**
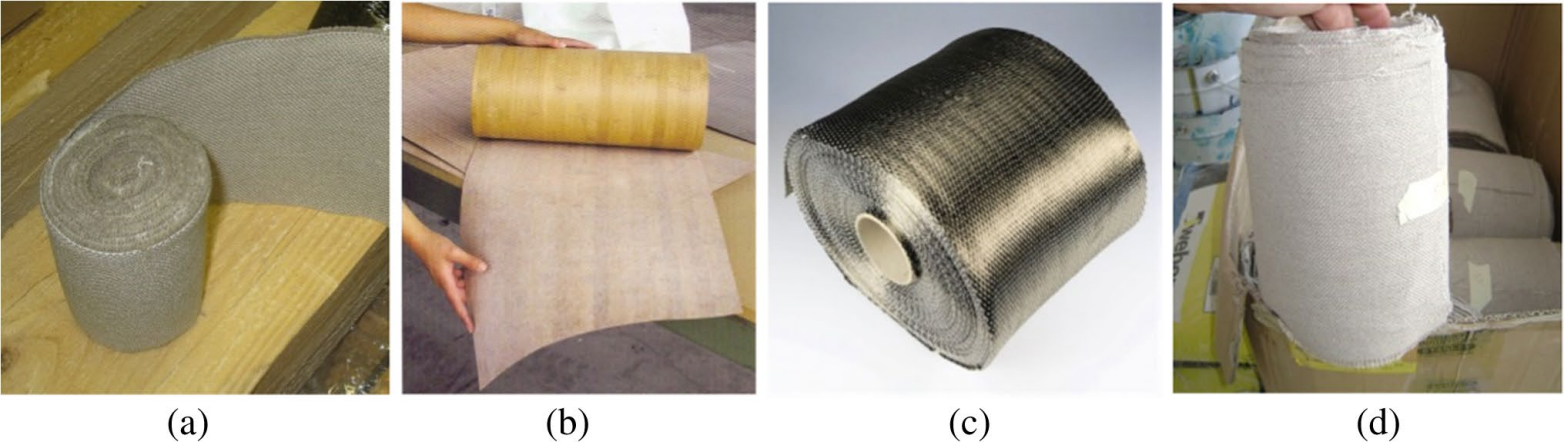
Natural fiber cloths: **a** flax, **b** bamboo, **c** basalt, **d** hemp

**Fig. 7:**
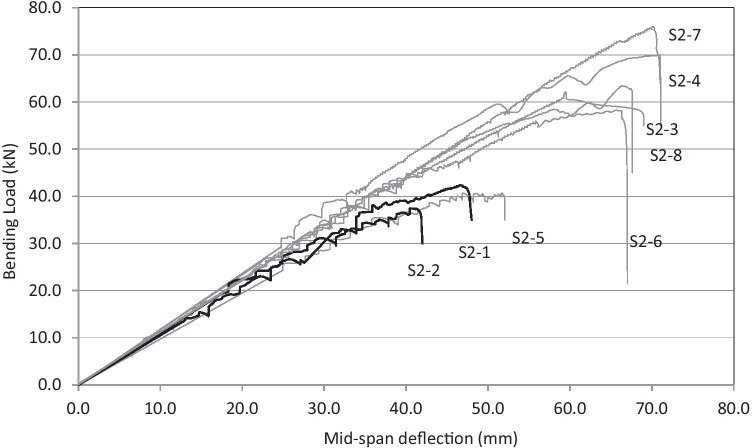
Bending test results of low-quality timber beams (dimensions 200 × 200 × 4000 mm) reinforced with natural fibers: S2-1 and S2-2 unreinforced; S2-3, S2-7, and S2-8, flax fiber reinforcement; S2-4, S2-5, and S2-6 basalt fiber reinforcement

**Fig. 8:**
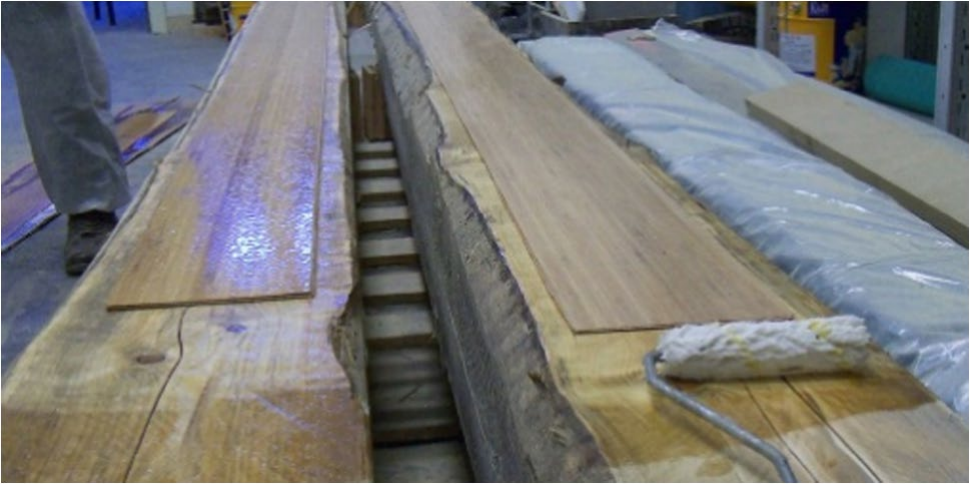
Reinforcement of timber beams with bamboo fibers: aesthetics impact is very low

Reinforcement of timber structures with unidirectional or bidirectional fiber sheets or cloths can be also highly beneficial, for example, to improve the bending capacity of timber beams, as fibers can reduce the weakling effect of natural defects in timber (knots, grain deviation, splits, etc.). The use of composite materials, bonded with resins on the beam tension side, is especially good when considered in terms of compatibility of their properties. This would be further confirmed if the fiber-reinforced composite consists of ecologically sustainable natural materials (Fig. 6, Table 2). The most common natural fibers used in wood reinforcement are basalt, hemp, flax, and bamboo fibers which can be successfully applied with any kind of wood such as solid wood, LVL (laminated veneer lumber), and glulam. Experiments were carried out on LVL reinforced with different types of fibrous composites (Speranzini and Tralascia, 2010). Test results demonstrate significant increments in the bending capacity of reinforced timber beams (Fig. 7) compared to unreinforced ones: capacity of the beams reinforced with flax fiber was only 15% smaller than the ones reinforced with carbon fibers. The increment in capacity load is maximum for beams reinforced with carbon fibers (approximately 42.3%, compared to unreinforced beams) and minimum in the case of reinforcement with basalt fibers. Among natural fibers, flax reinforcement demonstrated to be able to produce a significant increment in bending capacity, while bamboo fiber has a very low impact in terms of aesthetics given its resemblance to solid timber (Fig. 8).

**Table 2:** Physical and mechanical properties of natural and artificial fibers

	Diameter (µm)	Density (g/cm^3^)	Tensile strength (MPa)	Elongation at failure (%)	Young’s modulus (GPa)
Cotton (*Gossypium* sp.)	12–35	1.5–1.6	280–600	7–8	5.5–12
Juta (*Corchorus capsularis*)	5–25	1.3	390–750	1.5–1.8	25–27
Hemp (*Cannabis sativa*)	22–25	1.4	550–900	2–3	50–70
Flax (*Linum usitatissimum*)	10–80	1.4	500–1400	2–3	50–70
Sisal (*Agave sisalana*)	100–300	1.45	500–650	2–2.5	9–22
Bamboo* (*Phyllostachys pubescens*)	12–15	-	550–600	1.6–1.8	20–40
Basalt	13	2.6–2.7	2800–3500	2.3–2.6	70–90

*outer part of the culm

An interesting experimental campaign which examines the bending reinforcement of 20 fir wood rafters (40 × 50 × 1000 mm) and 25 beams (200 × 200 × 4000 mm) is presented in Borri et al. (2013). Rafters and beams were reinforced using hemp, flax, basalt, and bamboo sheets. Natural fiber reinforcement was applied to timber beams of low and good strength grade. Good strength grade timber beams did not exhibit high increases in the load-carrying capacity when reinforced with natural fibers. Particularly unsatisfactory results were found for reinforcement with bamboo fibers. For low-grade timber beams, reinforcement with basalt fibers produced significant increments in bending capacity, also improving the post-peak response by increasing the ductility. Reinforcement with flax fibers also resulted effective, as it produced improvement in flexural stiffness and capacity.

The main conclusion is that the world of natural fibers is complex and variegated: these fibers can exhibit very diverse properties in terms of mechanical characteristics, long-term behavior, and durability. However, the use of sustainable raw materials helps to protect ecosystems by reducing emissions and the unsustainable exploitation of raw material feedstocks, and a careful selection of the natural fibers to be used in engineering applications can often meet the needs in terms of mechanical properties.

With regard to reinforcement of masonry walls, mortar coatings reinforced with natural fibers represent a sustainable alternative for the retrofit of historic masonry buildings. This a development of the FRCM method described in the “FRCM” section where artificial fibers are replaced with natural ones. In Olivito et al. (2014), sisal and flax fibers were used to reinforce masonry walls, and test results showed significant increases in strength and stiffness of reinforced walls. The authors also considered the fibers’ durability and conducted aging tests on a natural fiber-reinforced lime mortar mix.

**Fig. 9:**
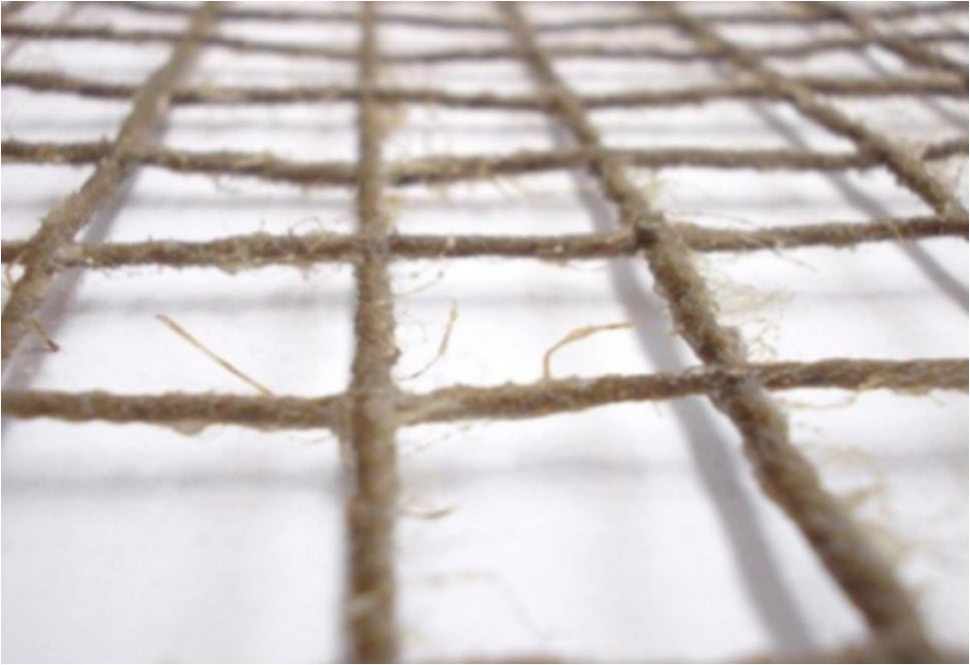
The grid of hemp fibers: this was embedded into a 15-mm lime (pozzolanic) coating (Menna et al. 2015)

In a study by Menna et al. (2015), masonry walls were strengthened using a grid of hemp fibers embedded into a 15-mm lime (pozzolanic) coating (Fig. 9). The method is very similar to the one shown in Fig. 4a. Shear test method was used for these walls, and the results were compared with unstrengthened walls: it was observed an increase in ductility and in shear capacity by a factor of 5.

Natural fiber-reinforced mortar coatings can be also used to enhance the out-of-plane capacity of wall panels. A 20-mm coating (single-sided reinforcement) of sisal fibers and rice straw was employed in Qamar et al. (2018), demonstrating significant improvements in terms of capacity, ductility, and stiffness.

Besides its ability to sustain loads, natural fiber-reinforced masonry and timber are also required to be durable, and this represents the main area of research in the future. Because of their organic structure, natural fibers can be attacked by biotic agents (fungi, insects, bacteria etc.) producing mechanical degradation.

#### Cross-laminated timber

The use of timber products in constructions is undoubtedly one of the most interesting solutions given the characteristics of timber in terms of natural carbon sink and renewable material. These characteristics and properties have made timber-based products popular in new constructions: the technical term green construction often refers to timber buildings. The use of timber in construction is very old and dates back many millennia, but in the twentieth century, especially in Europe, timber has been gradually displaced by other construction materials, such as steel and reinforced concrete.

In conservation engineering, a similar process occurred: timber roofs and floors (typically king post trusses in religious and public buildings, timber beam floors, wooden boards with structural purpose over the timber beams in private residences) have been demolished and replaced with RC floor and steel beams.

At the beginning of the 1990s, the scientific community working in the area of conservation engineering in seismic regions, started discussing the important limitations of this method: the removal of old timber structural members in historic masonry constructions and their replacement with RC ones often had devastating consequences on the structural response of these old buildings under the seismic actions. Not only the weight density of timber is several times smaller than concrete (400–600 kg/m^3^ and 2500 kg/m^3^, respectively) with the evident negative consequences in terms of increased seismic forces, given their inertial characteristics, but also RC member exhibits low deformation capacity and subsequently low energy dissipation compared to timber elements. The 1997 Central Italy, 1999 Athens, and 1999 Izmit destructive earthquakes clearly demonstrated the increased seismic vulnerability of masonry buildings where timber floors/roofs had been previously replaced with RC ones.

**Fig. 10:**
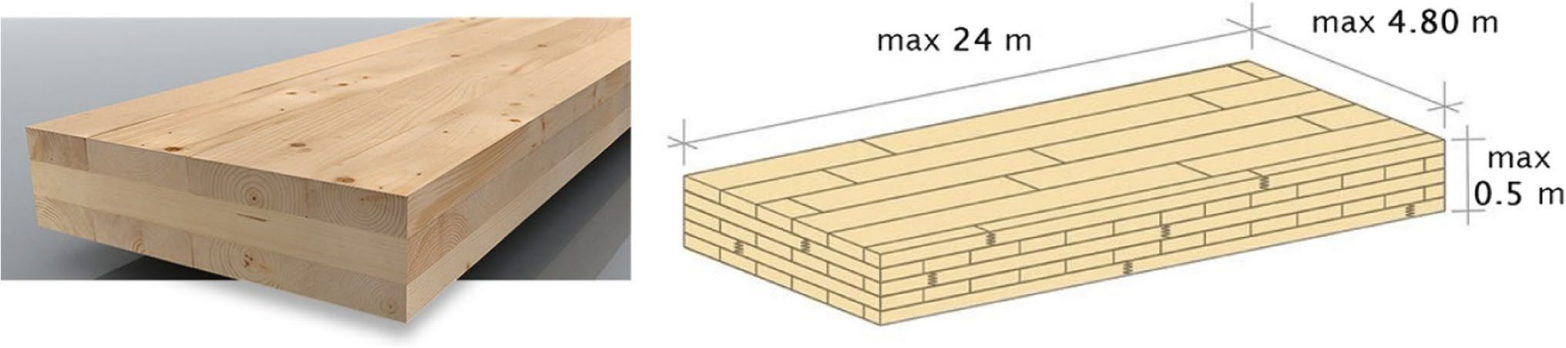
CLT is made from gluing together layers of selected solid-sawn lumber; thus, the effect of timber defects (knots, grain deviation, shakes, etc.) is highly reduced. Very large structural member (up to 24 × 4.8 × 0.5 m) can be fabricated in CLT

**Fig. 11:**
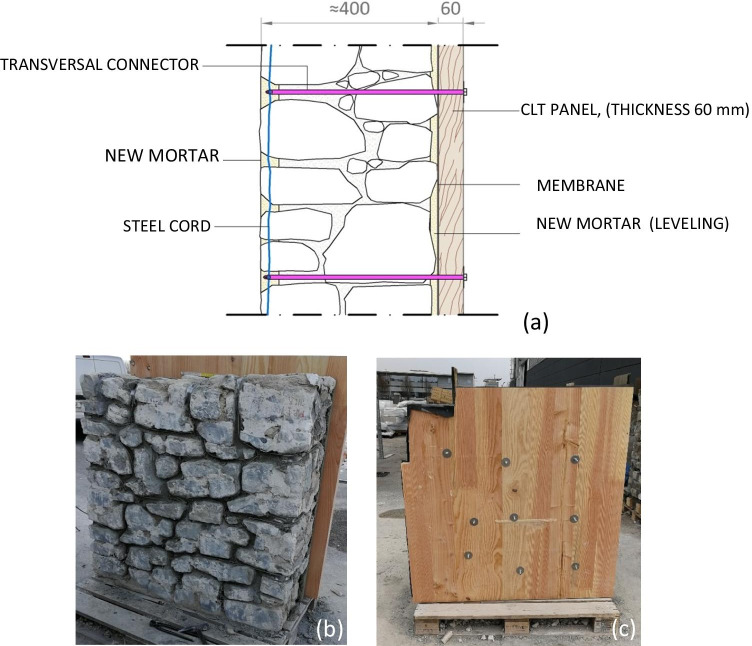
The use of CLT panels for shear reinforcement of walls: **a** detail of the wall section; **b** the “outdoor” wall face reinforced with steel cords embedded in the mortar joins, to preserve the fair face aspect of the masonry; **c** the “indoor” wall face with the CLT panel screwed to the masonry (Borri et al. 2020)

The previous research activities related to the use of CLT (Fig. 10) to old masonry structures have been focused both on theoretical and experimental aspects (Parisi and Piazza 2015a, 2015b; Giongo et al. 2017; Guo et al. 2017; Unuk et al. 2019). A limited amount of experimental work has been invested into testing of structural masonry under lateral shear loading. In 2020, Borri et al. (2020) carried out an interesting experimental work related to the use of CLT wall panels applied, like a jacketing, to the indoor face of stone masonry. To prevent the problems resulting from wetting and humidity on the CLT, the outdoor face wasn’t reinforced with CLT panels, but using steel cords embedded into the mortar joints. The final result (Fig. 11) is a combined reinforcement (CLT and steel cords) able to increase the lateral load capacity up to 100%, highly improving the thermal and acoustic efficiency of the building envelope.

**Fig. 12:**
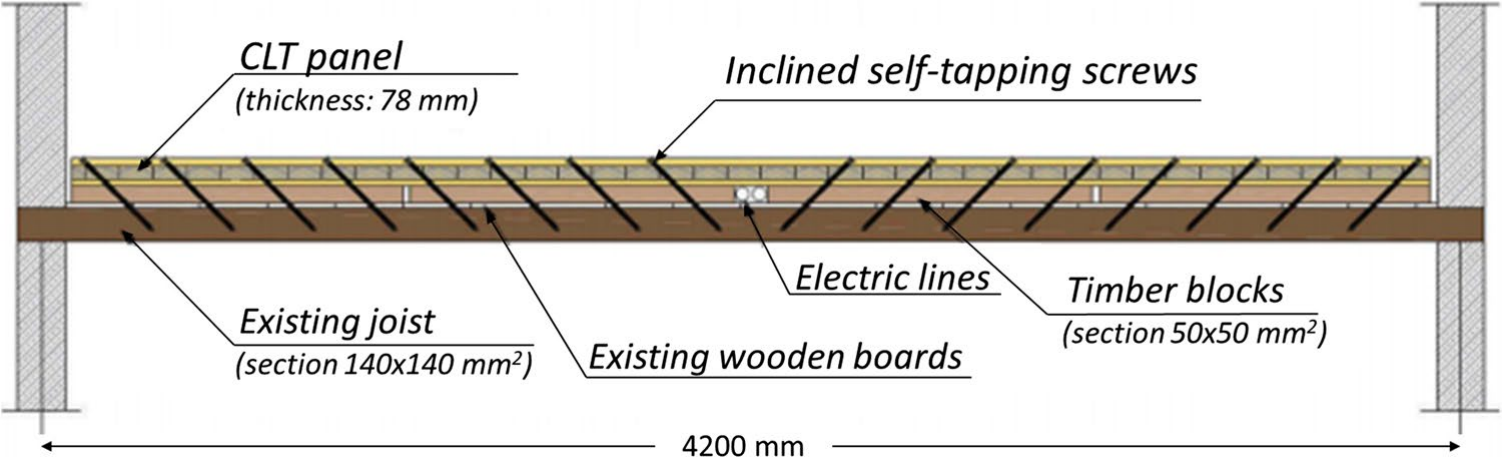
Combined shear and bending reinforcement of old timber floors: CLT panels are applied over the timber joists using inclined self-tapping screws (Roensmaens et al. 2018)

The reinforcement of old timber floors with CLT panels is another interesting application (Soriano et al. 2016; Gubana and Melotto 2018; Loss et al. 2018; Roensmaens et al. 2019; Rizzi et al. 2019; Longarini et al. 2020). Not only the unsatisfactory bending capacity, but also the low shear stiffness is an important problem for structural engineers dealing with rehabilitation of historic buildings. The low shear stiffness is particularly problematic for buildings located in seismic prone areas because the seismic forces cannot be distributed among the underlying masonry walls. This “lateral load distribution” is beneficial during seismic events because it reduces load concentration on the walls, especially the ones loaded perpendicularly to the direction of the seismic force (producing out-of-plane rocking), which are prone to collapse when struck by an earthquake. Roensmaens et al. (2018) proposed the use of CLT panels connected to the existing joists with inclined self-tapping screws, as shown in Fig. 12.

**Fig. 13:**
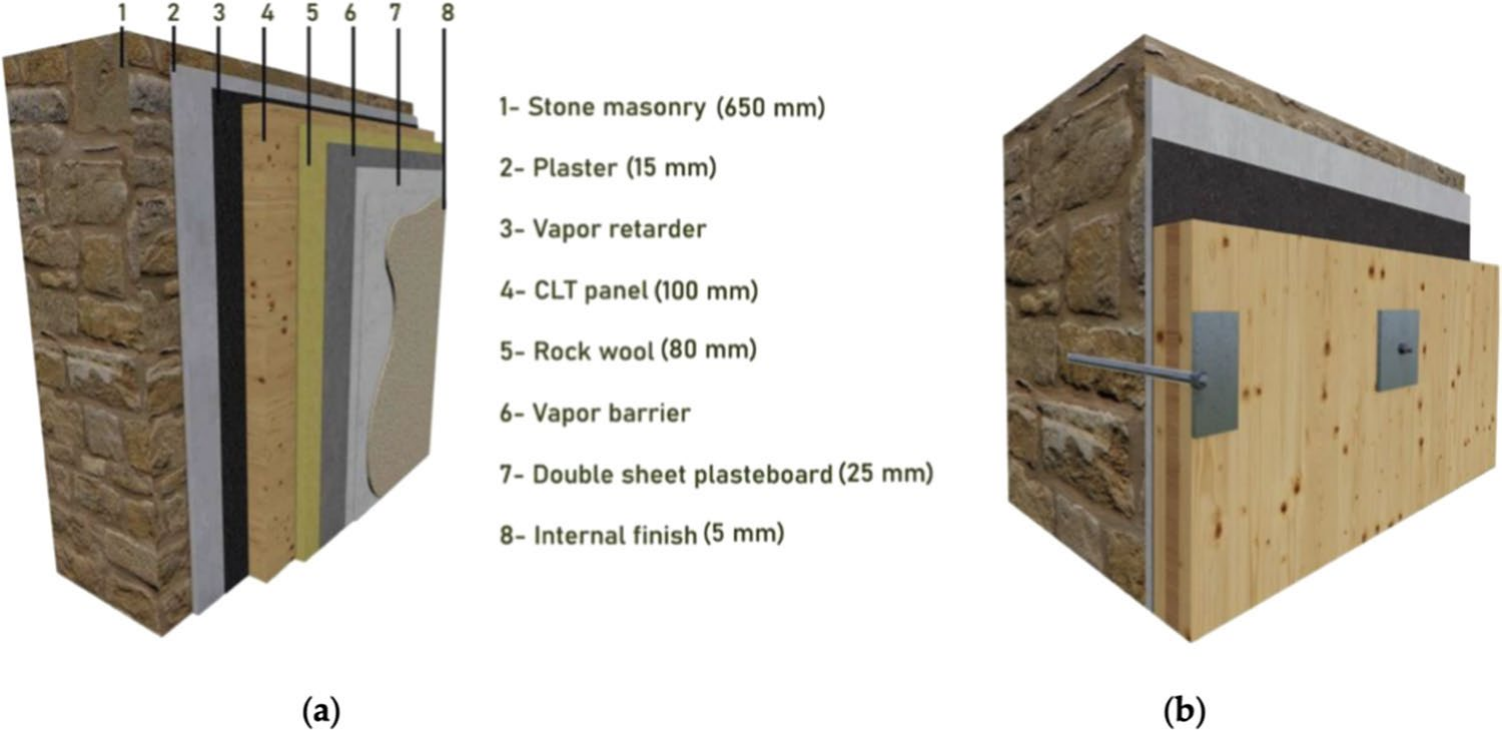
The method used in Valluzzi et al. (2021) for shear reinforcement of masonry

Riccadonna et al. (2019) conducted an experimental investigation on the use of timber panels to masonry wall dry connections under static and seismic shear loading conditions. Brickwork and stone masonry walls were reinforced using three different timber panels (spruce CLT, spruce LVL, and beech LVL) which were selected for the campaign. A similar solution was recently studied by Valluzzi et al. (2021) by proposing an integrated intervention approach able to preserve the external (outdoor) masonry face and to provide a new structural system for the inside one. The authors used CLT panels, thanks to their lightweight, high stiffness, and good hygrothermal characteristics, coupled with a vapor membrane and a rockwool panel (Fig. 13), connected transversally with bars.

The results of these experimental and numerical studies clearly demonstrated that important potentialities exist for the use of CLT in conservation engineering. However, two significant limitations have been highlighted: the connection of CLT wall panels to masonry, using steel rods/screws, can be ineffective due to embedment phenomena of the steel connectors when loaded in shear and the buckling instability of the CLT panels in compression (especially when the thickness is small). Furthermore, particular care should be used to protect the CLT from moisture penetration.

## Conclusions

Traditional masonry buildings, often dated back to the medieval times, are already good examples of sustainability in terms of environmental, economic, and social characteristics. Their re-use contributes to the reduction of the need for new constructions, water and energy consumption, and waste production and preserves the integrity of the soils, as a small quantity of aggregates is necessary in construction, and contributes to protect natural and man-made historic landscapes. Furthermore, old constructions are typically less dispersed as they are nucleated, and this causes a positive reduction in transport demand and fossil fuels.

Finally, building conservation has important cultural and social benefits as it promotes social identity, integration (e.g., cross-generation), and tourism and represents a tangible connection to the past, linking the new generations to their common past. Nevertheless, the structural behavior of historic construction is often unsatisfactory, especially when exposed to the loads induced by earthquakes, flooding, and other man-made and natural hazards. This can highly complicate their re-use and conservation.

Old constructions are typically made of stone or brick masonry (walls, vaults, and pillars) and timber (floors and roofs). In this paper, different sustainable retrofitting methods and materials have been described and discussed. From the results of this review study on the structural behavior of masonry members reinforced with sustainable materials or methods, the following conclusions can be drawn:

- According to the scientific literature, composite materials, made of carbon or glass fibers, are more effective for reinforcement of masonry structures, compared to natural fibers. However, due to the challenges of petroleum-based products (carbon, epoxies, etc.), natural fibers are an interesting alternative and dependent on renewable sources. These are also cost-effective, light, eco-friendly, and sustainable since they can be sourced from plants or animals. Their mechanical properties can be sometimes comparable to traditional composite material, and their application can be effective for a structural reinforcement of masonry and timber buildings.
- Fiber-reinforced cementitious mortars: the use of artificial and natural composite grids embedded into an inorganic matrix (a cement or, better, a lime coating) represents an interesting alternative to the use of petroleum-based organic resins (epoxy, polyester, etc.). Furthermore, an inorganic matrix is much more chemically compatible with historic masonry materials (stone, brick, lime) than an organic resin. The “reversibility” (minimal intervention and reversibility are key tenets of masonry heritage conservation) is typically possible by using FRCMs. Several research studies have demonstrated the efficiency of these retrofits for shear reinforcement, wall-to-wall connections, vaults, and arch reinforcement in masonry constructions.
- Cross-laminated timber: this green timber-based product exhibits very good mechanical properties and excellent characteristics for thermal and acoustic insulation of masonry buildings. In this paper, we have briefly reviewed two interesting applications of CLT in conservation engineering: the combined shear-bending reinforcement of timber floor and the shear reinforcement of masonry walls. Both of them produced significant improvement of the structural capacity of the reinforced members. The low weight of CLT applications is also particularly interesting in earthquake engineering given the inertial characteristics of the seismic forces.

## Data Availability

All data generated or analyzed during this study are included in this published article and in the referenced articles.
